# New ND-FISH-Positive Oligo Probes for Identifying *Thinopyrum* Chromosomes in Wheat Backgrounds

**DOI:** 10.3390/ijms20082031

**Published:** 2019-04-25

**Authors:** Wei Xi, Zongxiang Tang, Shuyao Tang, Zujun Yang, Jie Luo, Shulan Fu

**Affiliations:** 1 Provincial Key Laboratory of Plant Breeding and Genetics, Sichuan Agricultural University, Wenjiang, Chengdu 611130, Sichuan, China; xiwei923700915@163.com (W.X.); zxtang@sicau.edu.cn (Z.T.); tangshuyao705708@sina.com (S.T.); luojie2010@sina.com (J.L.); 2 College of Agronomy, Sichuan Agricultural University, Wenjiang, Chengdu 611130, Sichuan, China; 3 Center for Informational Biology, University of Electronic Science and Technology of China, Chengdu 610054, Sichuan, China; yangzujun@uestc.edu.cn

**Keywords:** wheat, *Thinopyrum*, chromosome, ND-FISH, oligo probe

## Abstract

*Thinopyrum* has been widely used to improve wheat (*Triticum aestivum* L.) cultivars. Non-denaturing fluorescence in situ hybridization (ND-FISH) technology using oligonucleotides (oligo) as probes provides a convenient and efficient way to identify alien chromosomes in wheat backgrounds. However, suitable ND-FISH-positive oligo probes for distinguishing *Thinopyrum* chromosomes from wheat are lacking. Two oligo probes, Oligo-B11 and Oligo-pThp3.93, were designed according to the published *Thinopyrum ponticum* (*Th. ponticum*)-specific repetitive sequences. Both Oligo-B11 and Oligo-pThp3.93 can be used for ND-FISH analysis and can replace conventional GISH and FISH to discriminate some chromosomes of *Th. elongatum*, *Th. intermedium*, and *Th. ponticum* in wheat backgrounds. The two oligo probes provide a convenient way for the utilization of *Thinopyrum* germplasms in future wheat breeding programs.

## 1. Introduction

*Thinopyrum intermedium* (*Th. intermedium*), *Thinopyrum ponticum* (*Th. ponticum*) and *Thinopyrum elongatum* (*Th. elongatum*) are important reservoirs of elite genes for wheat (*Triticum aestivum* L.) breeding programs. Genomic in situ hybridization (GISH) and fluorescence in situ hybridization (FISH) technologies can be used to differentiate and localize *Th. intermedium*, *Th. ponticum* and *Th. elongatum* chromosomes in wheat backgrounds [1,2,3,4,5,6,7,8]. However, GISH and FISH are time-consuming because of the preparation and labeling of probe sequences, and denaturing of the probes and chromosomes [9,10]. Oligonucleotide (oligo) probes combined with non-denaturing fluorescence in situ hybridization (ND-FISH) can be used to discriminate alien chromosomes in wheat backgrounds conveniently [10,11]. Suitable ND-FISH-positive oligo probes for distinguishing *Thinopyrum* chromosomes from wheat are lacking. In this study, *Thinopyrum* chromosome-specific oligo probes were developed.

## 2. Results

### 2.1. Production of Thinopyrum Chromosome-Specific Oligo Probes

*Th. ponticum* species-specific repetitive sequences B11 [3] and pThp3.93 [6] were aligned using the DNAMAN (Ver. 4.0, Lynnon Corp., Quebec, QC, Canada). The sequence of pThp3.93 has a 59.78% similarity with the 1138–1622 bp segment of the reverse_complement B11 sequence. The 1304–1348 bp segment of the reverse_complement B11 sequence and the 158–216 bp segment of the pThp3.93 sequence were used as oligo probes, and were named as Oligo-B11 and Oligo-pThp3.93, respectively. The two oligo probes are listed in Table 1.

### 2.2. ND-FISH Analysis Using Oligo-B11 and Oligo-pThp3.93

**Figure 1:**
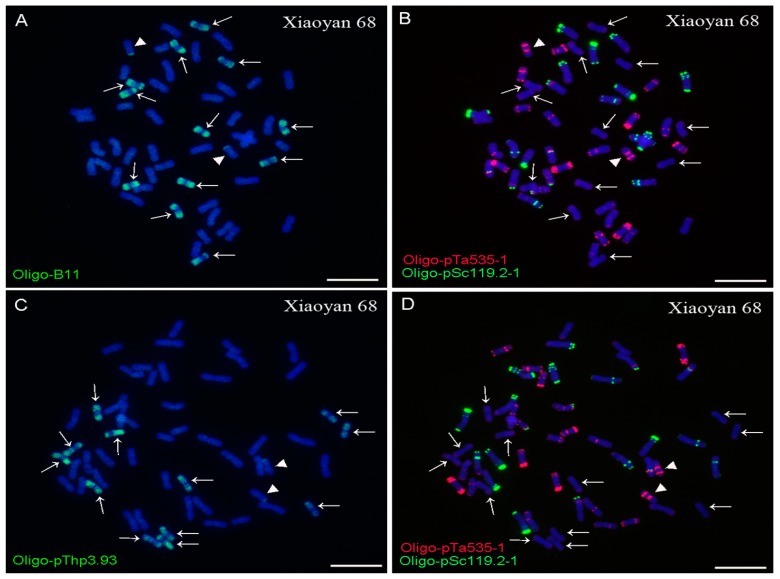
ND-FISH analysis of root tip metaphase chromosomes of Xiaoyan 68 using the Oligo-B11 (green), Oligo-pThp3.93 (green), Oligo-pTa535-1 (red), and Oligo-pSc119.2-1 (green) as probes. (**A**) and (**B**) are the same cell, and (**C**) and (**D**) are the same cell. Arrows indicate the *Th. ponticum* chromosomes that carry signals of Oligo-B11 (**A**) and Oligo-pThp3.93 (**C**). Triangles indicate the wheat-*Th. ponticum* translocation chromosomes TTh-4DS·4DL. Chromosomes were counterstained with DAPI (blue). Scale bar: 10 μm.

**Figure 2:**
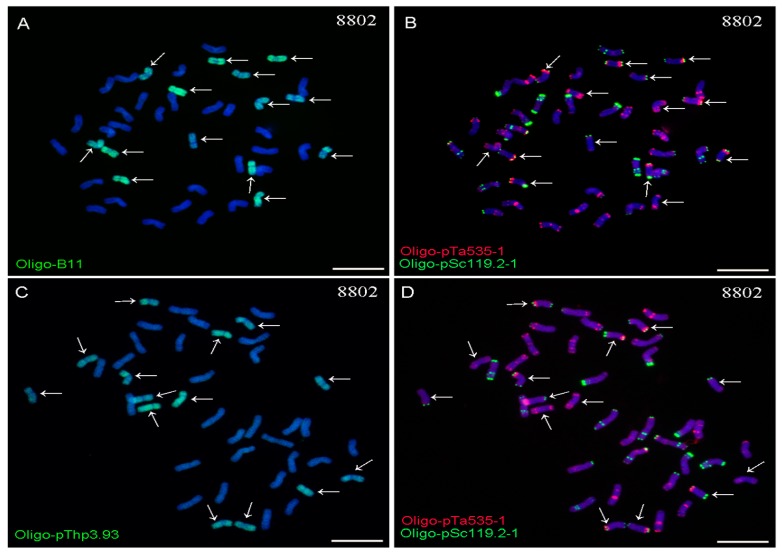
ND-FISH analysis of the root tip metaphase chromosomes of 8802 using the Oligo-B11 (green), Oligo-pThp3.93 (green), Oligo-pTa535-1 (red) and Oligo-pSc119.2-1 (green) as probes. (**A**) and (**B**) are the same cell, and (**C**) and (**D**) are the same cell. Arrows indicate the *Th. elongatum* chromosomes that carry signals of Oligo-B11 (**A**) and Oligo-pThp3.93 (**C**). Chromosomes were counterstained with DAPI (blue). Scale bar: 10 μm.

The Oligo-B11, Oligo-pThp3.93, Oligo-pSc119.2-1, and Oligo-pTa535-1 probes combined with the ND-FISH assay could distinguish *Thinopyrum* chromosomes from wheat in Xiaoyan 68, 8802, Xaioyan 7430, and Zhong 3. Both Oligo-B11 and Oligo-pThp3.93 probes produced dispersed signal patterns on 12 and 14 chromosomes in Xiaoyan 68 and 8802, respectively (Figure 1 and Figure 2). A pair of wheat-*Th. ponticum* translocation chromosomes TTh-4DS·4DL in Xiaoyan 68 could also be detected by Oligo-B11, Oligo-pThp3.93, Oligo-pSc119.2-1, and Oligo-pTa535-1 (Figure 1).

**Figure 3:**
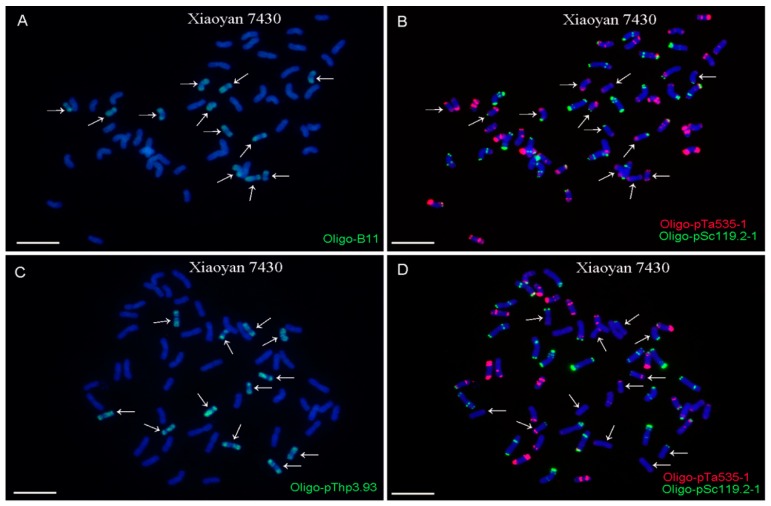
ND-FISH analysis of the root tip metaphase chromosomes of Xiaoyan 7430 using the Oligo-B11 (green), Oligo-pThp3.93 (green), Oligo-pTa535-1 (red) and Oligo-pSc119.2-1 (green) as probes. (**A**) and (**B**) are the same cell, and (**C**) and (**D**) are the same cell. Arrows indicate the *Th. ponticum* chromosomes that carry signals of Oligo-B11 (**A**) and Oligo-pThp3.93 (**C**). Chromosomes were counterstained with DAPI (blue). Scale bar: 10 μm.

**Figure 4:**
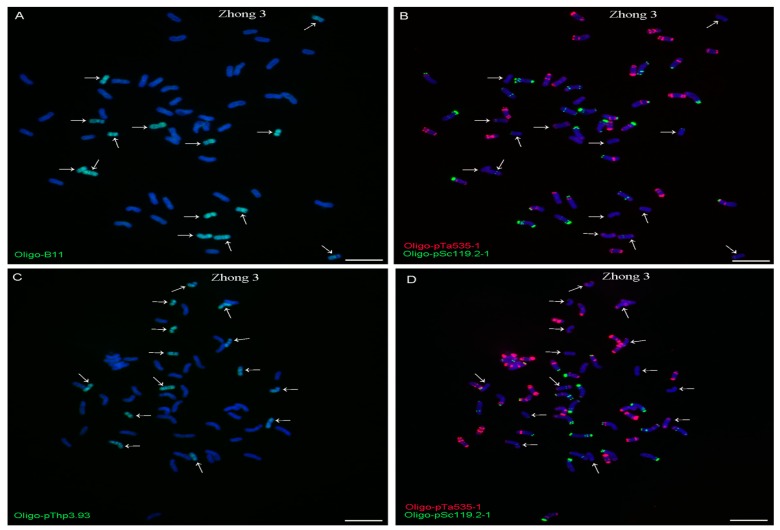
ND-FISH analysis of the root tip metaphase chromosomes of Zhong 3 using the Oligo-B11 (green), Oligo-pThp3.93 (green), Oligo-pTa535-1 (red) and Oligo-pSc119.2-1 (green) as probes. (**A**) and (**B**) are the same cell, and (**C**) and (**D**) are the same cell. Arrows indicate the *Th. intermedium* chromosomes that carry signals of Oligo-B11 (**A**) and Oligo-pThp3.93 (**C**). Chromosomes were counterstained with DAPI (blue). Scale bar: 10 μm.

The Oligo-B11 and Oligo-pThp3.93 probes also produced whole-chromosome signal patterns on 12 and 14 chromosomes in Xiaoyan 7430 and Zhong 3, respectively (Figure 3 and Figure 4). Therefore, both of the ND-FISH-positive Oligo-B11 and Oligo-pThp3.93 probes produced whole-chromosome signal patterns on 12, 14, 12, and 14 chromosomes in Xiaoyan 68, 8802, Xaioyan 7430, and Zhong 3, respectively. Additionally, no obvious signals of Oligo-B11 and Oligo-pThp3.93 were observed on wheat chromosomes in the materials used in this study (Figure 1, Figure 2, Figure 3 and Figure 4).

## 3. Discussion

### 3.1. Using Oligo Probes and ND-FISH Assay to Identify Alien Chromosomes

Since Cuadrado et al. used the ND-FISH assay to detect plant telomeres [12], this technology has been widely used to analyze chromosomes of Triticeae species because of its convenience and high efficiency [4,10,11,13,14,15,16,17,18,19,20,21,22,23,24,25,26]. Using oligo probes combined with the ND-FISH assay, rye (*S. cereale*) and *Dasypyrum villosum* chromosomes can be effectively and accurately distinguished from common wheat chromosomes [4,10,11]. In addition, two ND-FISH-positive oligo probes, Oligo-B and Oligo-D, can replace multicolor GISH to identify wheat A-, B-, and D-genome chromosomes [23]. During the ND-FISH procedure, oligo probes can be commercially synthesized and the denaturing of probes and chromosomes is not necessary [4,10,11,23]. Therefore, compared with GISH and FISH, ND-FISH analysis with suitable oligo probes can efficiently distinguish the chromosomes between two distant genera and within the same genus of the Triticeae tribe.

### 3.2. ND-FISH-Positive Oligo Probes for Identifying Thinopyrum Chromosomes

For a successful ND-FISH assay, finding suitable oligo probes is the key initial step. However, the necessary ND-FISH-positive oligo probes for distinguishing *Thinopyrum* chromosomes in wheat backgrounds are still lacking. Some disease-resistance and stress-resistance genes from *Thinopyrum* have been introduced into wheat backgrounds [1,2,3,4,5,6,7,8,18,27]. Now, conventional GISH and FISH are the main methods to distinguish and localize *Thinopyrum* chromosomal segments in wheat backgrounds [1,2,3,4,5,6,7,8,18,27]. In this study, two suitable ND-FISH-positive oligo probes, Oligo-B11 and Oligo-pThp3.93, were designed based on *Th. ponticum* species-specific repetitive sequences [3,6]. Both of these oligo probes produced whole-chromosome signal patterns on 12, 14, 12, and 14 chromosomes in Xiaoyan 68, 8802, Xaioyan 7430m and Zhong 3, respectively (Figure 1, Figure 2, Figure 3 and Figure 4). The numbers of the *Thinopyrum* chromosomes identified by Oligo-B11 and Oligo-pThp3.93 in each of these materials were consistent with those previously reported [1,6,28,29].

In addition, the Oligo-B11, Oligo-pThp3.93, and Oligo-pTa535-1 probes could also identify the TTh-4DS·4DL translocation chromosomes in Xiaoyan 68 (Figure 1). In the materials used in this study, the *Thinopyrum* chromosomes came from *Th. ponticum*, *Th. elongatum*, and *Th. intermedium* [1,6,28,29]. Therefore, Oligo-B11 and Oligo-pThp3.93 combined with the ND-FISH assay can replace conventional GISH and FISH to conveniently discriminate the *Th. elongatum*, *Th. intermedium*, and *Th. ponticum* chromosomes in Xiaoyan 68, 8802, Xaioyan 7430, and Zhong 3 from wheat chromosomes.

## 4. Materials and Methods

### 4.1. Plant Materials

Wheat-*Th. ponticum* partial amphiploids Xiaoyan 68 and Xaioyan 7430, hexaploid *Trititrigia* 8802, and wheat-*Th. intermedium* partial amphiploid Zhong 3 were kindly provided by Professor Fangpu Han, Institute of Genetics and Developmental Biology, Chinese Academy of Science, Beijing, China. The 8802 contains chromosomes of *Th. elongatum* [28].

### 4.2. Cytological Analysis

Root-tip metaphase chromosomes were prepared following the methods described by Han et al. [30]. Oligo-pSc119.2-1 and Oligo-pTa535-1 were also used in this study [31]. Oligo-B11, Oligo-pThp3.93, Oligo-pSc119.2-1, and Oligo-pTa535-1 probes were synthesized by Tsingke Biological Technology Co. Ltd. (Beijing, China). Both the Oligo-B11 and Oligo-pThp3.93 probes were 5′-end-labelled with 6-carboxyfluorescein (6-FAM), the Oligo-pTa535-1 probe was 5′-end-labeled with 6-carboxytetramethylrhodamine (TAMRA), and the Oligo-pSc119.2-1 probe was 5′-end-labeled with Cyanine Dye 5 (Cy5). The synthesized oligo probes were diluted by 1 × TE solution (pH 7.0). For Oligo-pSc119.2-1 and Oligo-pTa535-1, probe dilution and the probe amounts per slide were carried out according to the methods described by Tang et al. [31]. For Oligo-B11 and Oligo-pThp3.93, 100 μL 1 × TE was used to dissolve each 1OD probe. Then, the original solution was diluted five times and were used as the working solutions. Two × SSC and 1 × TE buffers (pH 7.0) were mixed as a 1:1 (volume) ratio. The target probes were added into the 2 × SSC + 1 × TE buffer and uniformly mixed. For each slide, 10 μL of probe mixture was used. When the probe mixture was dropped onto the cell spreads, the room temperature around the slide were kept at above 28 °C, then the slides were covered with glass coverslips and immediately put in a moist box that was incubated at 42 °C in advance, and then stored in the moist box at 42 °C for 1–2 h. After incubation, the slides were washed for 15–20 s in 2 × SSC at 42 °C. When the slide washing was completed, the slides were dried with a rubber suction bulb, and the slides were mounted with Vectashield mounting medium (Vector Laboratories, Burlingame, CA, USA) with DAPI (4′,6-diamidino-2-phenylindole). An epifluorescence microscope (BX51, Olympus Corporation, Tokyo, Japan) equipped with a cooled charge-coupled device camera operated with HCIMAGE Live software (Hamamatsu Corporation, Sewickley, PA, USA) was used to take images.

## 5. Conclusions

In conclusion, the Oligo-B11 and Oligo-pThp3.93 probes combined with the ND-FISH assay can replace GISH and FISH to conveniently discriminate the *Th. elongatum*, *Th. Intermedium*, and *Th. ponticum* chromosomes in Xiaoyan 68, 8802, Xaioyan 7430, and Zhong 3 from wheat chromosomes. Therefore, the two oligo probes provide a convenient way for the utilization of *Thinopyrum* germplasms in Xiaoyan 68, 8802, Xaioyan 7430, and Zhong 3 in future wheat breeding programs.

## Figures and Tables

**Table 1:** Oligonucleotide sequences of new oligo probes developed in this study.

Probe	Amount for Each Slide (ng/slide)	Oligonucleotide Sequence (5′-3′)
Oligo-B11	72.5–96.6	TCCGCTCACCTTGATGACAACATCAGGTGGAATTCCGTTCGAGGG
Oligo-pThp3.93	69.5–92.6	GGACTCCCACTAGATGTATCCGTCAAGGTGAATCCAGAGGAATCACCCTCGATGGCATT
